# Examining ecological validity in social interaction: problems of visual fidelity, gaze, and social potential

**DOI:** 10.1007/s40167-016-0041-8

**Published:** 2016-09-22

**Authors:** Arran T. Reader, Nicholas P. Holmes

**Affiliations:** 1 School of Psychology and Clinical Language Sciences, University of Reading, Earley Gate, Whiteknights Road, Reading, RG6 6AL UK; 2 School of Psychology, University of Nottingham, Nottingham, UK

**Keywords:** Social interaction, Ecological validity, Gaze, Visual fidelity, Social potential, Two-person

## Abstract

Social interaction is an essential part of the human experience, and much work has been done to study it. However, several common approaches to examining social interactions in psychological research may inadvertently either unnaturally constrain the observed behaviour by causing it to deviate from naturalistic performance, or introduce unwanted sources of variance. In particular, these sources are the differences between naturalistic and experimental behaviour that occur from changes in visual fidelity (quality of the observed stimuli), gaze (whether it is controlled for in the stimuli), and social potential (potential for the stimuli to provide actual interaction). We expand on these possible sources of extraneous variance and why they may be important. We review the ways in which experimenters have developed novel designs to remove these sources of extraneous variance. New experimental designs using a ‘two-person’ approach are argued to be one of the most effective ways to develop more ecologically valid measures of social interaction, and we suggest that future work on social interaction should use these designs wherever possible.

## Introduction

Social interaction is the combination of individual and joint behaviour that occurs between two or more individuals. It can be influenced by a wide range of variables such as gender, age, or nationality. Many of these variables are of interest to researchers, and we can refer to these as ‘interest variables’. On the other hand, there are a number of sources of variance often unaccounted for in the design and analysis of experiments. This review is not concerned with experimenter bias, or equipment errors, but the variance between naturalistic and experimental behaviour that occurs as a result of the experimental design itself (i.e., introduced as a direct result of the way in which we choose to study social interaction). We could call the sources of such variance ‘nuisance variables’.

Experiments in social interaction are designed in a number of different ways. For example, a single participant observer may be requested to respond to an actor, either in real life or on a screen. They may be asked to perform interactive actions or gestures, or simple finger movements. The tasks could be performed either separate to or in addition to neuroimaging. However, problems may arise in designs which are lacking social interactivity. Indeed, Risko et al. (2012) highlighted the fact that many studies of social interaction use stimuli that are “socially relevant”, but may be lacking in vital aspects of real life social interactions—particularly video stimuli. Risko et al. (2012) are not alone in their concerns (e.g., de Jaegher et al. 2010; Gregory et al. 2015; Hogenelst et al. 2015). Indeed, de Jaegher et al. (2010) emphasised that interaction in social cognition is not simply contextual (i.e., a way of framing two individuals’ actions), but a vital component of social cognitive processes. Ecologically invalid paradigms for examining interactive behaviours (such as those using a single participant responding to a video rather than an actual human being) are likely either to introduce ‘nuisance variance’, or to constrain behavioural variance unnaturally. Addressing this issue could help to unify methods and theory in the field and in this way advance our knowledge. This brief review considers three potential sources of nuisance variance—gaze, visual fidelity, and social potential—and suggests that two-person experimental designs may be an effective way to develop a more valid understanding of social interaction.

## The importance of interaction

In order to reduce the effects of nuisance variables in social interaction, we first need to recognise them, and measure the degree to which they might change the behaviour of interest. Only until relatively recently have some results revealed surprising variables that may influence social interaction—many related to common approaches that are taken towards experimentation.

### Visual fidelity

One factor that is common across different research areas is the use of video stimuli in place of real two-person interactions. Indeed, video stimuli are effective and highly controllable methods of presenting information to participants. Risko et al. (2012), however, suggested that some socially relevant tasks may suffer under the influence of “reel” (i.e., recorded) versus “real” (i.e., live) stimuli. Taken at face value, this is not surprising. Social interaction typically takes place in scenarios that feature more than one individual reacting to one or more others in a dynamic, instantaneous fashion. Even just the *potential* for social interaction in an experiment can alter behaviour in observable ways (Laidlaw et al. 2011), and this is discussed in more detail below. One could argue that video stimuli reduce social interaction to the level of social *observation*. Any differences between video and real life interaction could comprise the most prominent potential causes of nuisance variance, since video stimuli are so widely used. Unfortunately, little work has been done to examine how this nuisance variable might affect participant behaviour in experiments on social interaction.

The potential effects of using video stimuli in socially relevant scenarios were, perhaps surprisingly, revealed in neurophysiological experiments before they were revealed through behavioural testing. Järveläinen et al. (2001) found reduced primary motor cortex activation (as measured by magnetoencephalography) during the observation of video versus real life hand movements (see Ruysschaert et al. 2013 for a similar effect in infants). Järveläinen et al. (2001) posited that ecological validity was important in their findings. Their real life action condition was more representative of the way in which actions are observed in daily life and therefore more likely to increase participant interest, attention, or motivation. However, such results suggesting differences in behavioural responses between real and video stimuli are rare.

Recently, Reader and Holmes (2015) tested whether transitive imitation accuracy varied between face-to-face and video feedback. By using a two-person design along with motion-tracking, we tested participants’ imitation ability when they were seated opposite an actor, or when their only direct interaction with the actor was through a live video feed (although both participants were in the same room, and aware of each other’s presence). We found that task-specific imitation (i.e., copying the movement of objects to a series of locations) was significantly worse in video feedback conditions. Accuracy in a 3D task, measured through the correlation between actor and imitator, was reduced by presenting the stimuli in 2D. These results indicated that social interactive abilities such as imitation may be undermined by video stimuli. We suggested that the most likely cause of this effect was the difference in visual fidelity between the two conditions. Since the treatment of 3D and 2D visual information by the visual system is different (Patterson 2009), with, for example, more cues to depth in 3D than 2D, it follows that the visuomotor responses to such stimuli might be different, particularly if the visual information guiding the action is sub-optimal. Furthermore, higher-level processes (i.e., attention) that are reliant on low-level visual cues might also be negatively influenced. Importantly, this might mean that measures of social interaction using video stimuli are not a wholly accurate measure of participants’ true ability. It is also worth noting that results such as ours may also have been due to differences in gaze behaviour between video and face-to-face feedback conditions.

### Gaze

Vision is an important aspect of social interaction, and visual information is likely impoverished in single-participant designs (Skarratt et al. 2012). The gaze of an observer may alter depending on the context in which they are placed—in the lab or in real life. For example, Foulsham et al. (2011) used a mobile eye-tracker to examine eye movements in real life scenarios, and then compared them to the eye movements of participants watching a video recorded from the point of view of the original, moving observer. They found distinct differences in gaze behaviour (i.e., eye and head movements) between the lab and real life scenarios, suggesting that gaze as measured in laboratory settings may not be indicative of real life behaviour. Similarly, Pönkänen et al. (2010) found greater face-sensitive event-related potentials during direct gaze between real life individuals, compared to pictures of individuals. In particular their results suggested that early processing of face-related information is enhanced when the observed person’s face and eyes are seen directly rather than via video.

Similarly, the gaze of an individual that we are interacting with may be important. In social interactions, the gaze of an actor can provide a cue to attention for the observer (Friesen and Kingstone 1998), and another individual’s gaze can strongly influence where we direct our own gaze (Gallup et al. 2012). An experiment by Letesson et al. (2015) confirmed the importance of gaze in a social action execution task that results in a phenomenon known as ‘action priming’. Action priming is commonly thought of as the facilitation of an action following observation of a congruent action performed by a second person. This effect is typically considered in regards to body kinematics. Letesson et al. (2015) showed participants videos of people performing transitive (i.e., object-directed) actions. These videos varied in the availability of both gaze cues (in which the actor’s gaze was explicitly directed to an object), and grasp kinematic cues (in which the video actor reached-to-grasp a small or large object). The actor in the videos directed their gaze towards a small or large object, directed their gaze towards and reached-to-grasp the object, reached-to-grasp the object whilst their gaze was obscured, or performed no reach-to-grasp or gaze changes. The participants had to respond with a congruent (reach-to-grasp same size object) or incongruent (reach-to-grasp different size object) action. Their eye and hand movements were recorded throughout. The results suggested that gaze and grasp kinematic cues contributed differently to action priming, with gaze cues influencing the speed with which participants attended to the target (as measured using eye-tracking), and grasp kinematic cues influencing the accuracy of such attention. Such findings clearly indicate the importance of gaze cues in guiding an observer’s behaviour during social interaction, suggesting an interaction between gaze and body kinematics that may be undermined in certain experimental methods.

Gaze cues in real life (i.e., non-laboratory) interactions are likely to be dynamic and contextual. Gaze is not always controlled for in pre-recorded video stimuli (e.g., Campione and Gentilucci 2011; Fernando and Rob 2015), although some experiments on social interaction exclude the face (e.g., Hardwick et al. 2012; Mühlau et al. 2005; Naish et al. 2013), often to control for emotional content or intention. While excluding the face is appropriate for experiments involving body movements, it may be invalid to make claims regarding social interaction in general from these results. Gaze cueing that is not controlled for or specified could be a source of nuisance variance. As such, the results of Letesson et al. (2015) suggest that gaze and kinematics may be highly complementary aspects of social interaction. The separation of kinematic and gaze cues (as is common in many experiments) may have adverse effect on the validity of social interaction experiments if real social interaction is reliant on a combination of the two.

### Social potential

As we have seen, sources of nuisance variance in social interaction could be due to differences in visual fidelity or gaze behaviour. However, experimental paradigms that rely on testing a single individual may suffer from an effect of reduced ‘social potential’. That is, the potential for two-way social interaction is reduced, possibly resulting in differences in behaviour compared to that which may occur in real interactions.

Much has been said about the potential differences between acting in isolation and acting in a social context (e.g., Becchio et al. 2010), and it is well established that the intentions, actions, and location of another individual can affect the ways in which we interact with them (e.g., our action kinematics). For example, by changing the presence of an observer, along with the observer’s proximity and their ability to intervene in the participant’s action, Quesque et al. (2013) found that the kinematic parameters of participants’ object-directed actions (in particular preparatory actions) were significantly altered. This was true even in cases where it was impossible for the observer to directly influence the action outcome. These results imply that the mere presence of another individual can have important effects on behaviour. Reliance on single-participant designs may not, therefore, be adequate for testing social interaction—removing the potential for real interaction could introduce nuisance variance if there is an interaction between nuisance and interest variables. In particular, experimental power may be reduced in such instances.

In addition to this problem of absent interaction potential *between* an observer and an actor, there may also be differences dependent on the social potential *within* the observed stimuli. In a recent experiment, Aihara et al. (2015) applied transcranial magnetic stimulation (TMS) over the motor cortex of participants whilst they observed interactive behaviour between two individuals, or non-interactive behaviour featuring a single individual. They found increased corticospinal excitability (as measured by motor evoked potentials) during the observation of social interactions. Whilst there was no potential for participant interaction in this experiment, the results suggested that even relatively fundamental measures of neurophysiology are influenced by social potential within an observed stimulus. In particular, if we want to say more about participant responses to complex social scenarios involving the observation of more than two individuals, it may be necessary to provide participants with stimuli more representative of the naturalistic way in which we interact. Our social interaction is not just defined by our interactions with one other person, but also with *another person*’*s potential interactions with a third person*. Taken together, the results of Quesque et al. (2013) and Aihara et al. (2015) suggest that the social context of a particular scenario strongly influences physiology and behaviour, and that more could be done to develop the context of social interactions to move beyond the observation of single actor–object interactions.

## Improving methodology

The above experiments suggest that whilst a number of nuisance variables (differences in visual fidelity, gaze, and social potential) may influence results in studies of social interaction, a number of these could be controlled for through the presence of a second person with whom the participant can interact. This second person has been the topic of growing discussion (Schilbach 2010; Schilbach et al. 2013), and many differences between realistic and laboratory based social cognition could be seen as a symptom of the absence of this second person. For example, reduced visual fidelity in 2D versus 3D viewing, non-dynamic gaze cueing, and a lack of interaction potential all arise from the use of video stimuli in the testing of single participants. As such, it appears that there is a need to improve methodology by developing experiments that enable the participant to observe and interact with a second person. This theme continues in the following suggestions, and makes clear that two-person designs are possible in a number of different research areas and methodological approaches.

### Joint action and interpersonal coordination

Joint action (cooperative or competitive action towards a shared or individual goal) and interpersonal coordination (explicit or implicit co-movement in more than one individual) are important aspects of social interaction (Marsh et al. 2009), and studies in this area have done much to improve the study of social interaction. For example, Georgiou et al. (2007) examined the kinematics of participants’ reach-to-grasp actions when they were requested to grab an object under two different contexts: cooperation or competition. They found that the contexts resulted in differing kinematics, and a higher correlation between participants’ kinematics (time to maximum trajectory height and time of maximum grip aperture) during cooperation. Other research has revealed that cooperative joint actions are improved by participant movement synchrony (synchronous rocking in the case of Valdesolo et al. 2010), and altered by the role each participant is assigned (e.g., leader or follower, Sacheli et al. 2013). In addition to this, two-person type interactions in joint action research have recently developed our understanding of how group membership might alter participant behaviour (Aquino et al. 2015).

Interpersonal coordination has long made use of two-person paradigms, perhaps unsurprisingly. A review by Keller et al. (2014) highlights some of the work performed in this area, and emphasises how some new experimental paradigms (e.g., examining music ensembles) may provide a powerful balance between ecological validity and experimental control. Despite the progress made in joint action and interpersonal coordination, which maintain a high level of ecological validity and may avoid a number of nuisance variables, other topics have generally failed to embrace two-person designs. Research into subjects such as action observation and imitation could benefit greatly from a move towards the type of two-person paradigms used in joint action experiments, and help reduce nuisance variance stemming from invalid representations of variables such gaze or social potential.

### Virtual characters

Virtual reality (VR) and virtual characters provide other ways in which to increase the representativeness of the phenomena of interest whilst in the lab, and such methods have been found to be valid for a number of tasks (Bombari et al. 2015). Virtual characters might therefore provide a more valid way of testing social interaction, at least compared to static images or pre-recorded video stimuli. For example, Pan and Hamilton (2015) used a virtual character to measure automatic imitation in a more realistic context than similar previous studies, which generally relied on video presentation of simple finger movements. Automatic imitation describes the phenomenon in which participants respond faster to an imitative task than to a matched, non-imitative task, following action priming (the facilitation of movement following observation of a congruent movement). Automatic imitation has typically been examined by measuring reaction time to perform a key press whilst observing spatially congruent or incongruent finger movements on a screen. Pan and Hamilton (2015) moved beyond this common paradigm and asked participants to imitate a virtual character performing sequential hand-arm movements directed at three drums. This was compared with a control condition in which virtual balls performed the actions. Both of these conditions were tested with movements imitated in either a spatial (i.e., towards the same spatial location) or anatomical (i.e., towards the anatomically correct position as defined by the character’s anatomy) fashion. They found that automatic imitation was present for virtual characters but not for balls when imitation was in anatomical fashion. They also found that participants reacted quicker the more they felt that the character was human, once again highlighting the importance of social potential.

In another novel experiment, Sacheli et al. (2015b) requested participants to perform imitative or complementary joint actions with either a racial in-group or out-group virtual character. Their results suggested that visuomotor interference during joint action is modulated by racial bias. The results of Pan and Hamilton (2015) and Sacheli et al. (2015b) have provided a more realistic insight into well-established phenomena, allowing us to be more confident that previous results are in line with real life behaviour. Virtual characters have the benefit of being controllable, allowing experimenters to modulate various aspects of interest during testing. For example, one can alter the presence or absence of mimicry in a virtual character to change how the character is perceived by the observer (Bailenson and Yee 2005). Furthermore, virtual characters have the ability to reduce the variability of behaviour that can occur across trials which is inherent in two-person designs. Virtual characters may represent a compromise between full two-person interactions and fully controllable, pre-recorded stimuli.

### Neuroimaging

Though behavioural studies are useful, often we want to understand the neurophysiological basis of behaviour. Hence, we turn to neuroimaging. Social interaction studies using neuroimaging may struggle to maintain ecological validity, mainly due to the restricted scanning environment. It is difficult to test more than one person at a time, and participant movement is limited to prevent artefacts. However, some progress has been made in order to make more ecologically valid (i.e., two-person) neuroimaging a reality. In one early study of this kind, Decety et al. (2002) examined the neural mechanisms involved in reciprocal imitation using positron emission tomography (PET). By making use of mirrors and projectors, Decety et al. (2002) enabled participants to interact with various objects placed within grasping distance as they imitated and were imitated by an experimenter. A different approach was taken by Kokal et al. (2009), who used a custom response box that was magnetic resonance imaging (MRI) compatible. The participant and the experimenter (also in the scanner room) could then interact with the same response box in cooperative and non-cooperative conditions. In an experiment by Redcay et al. (2010), participants played simple cooperative games with an experimenter whilst observing the experimenter through a live video feed. The benefit of such a design is that it keeps participants still enough to be scanned, whilst ensuring that interaction is both dynamic and near real-time, and therefore more ecologically valid (despite still having to use video stimuli). In another neuroimaging experiment, this time looking at gaze, Cavallo et al. (2015) used a custom functional MRI (fMRI) setup that also allowed their participants to view another individual directly (via a mirror) providing a proof of principle that fundamental aspects of social interaction can be measured in a highly ecological manner within the scanning environment.

Whilst studies such as these inform us of the brain regions that might be involved in various social interaction tasks, neurostimulation can provide more cause-and-effect conclusions. In one example TMS was used to great effect with a virtual character (Sacheli et al. 2015a). Further development of neurostimulation methods alongside two-person or virtual character experimental designs would add greater depth to the growing research on neural aspects of social interaction by allowing us to examine cause-and-effect detail regarding certain brain areas and their role in realistic social interaction. The work of the above authors highlights interesting new approaches that provide ingenious ways to make neuroimaging in social interaction more ecologically valid.

## What next?

Ecologically valid paradigms are slowly but surely becoming more common, driven in part by an increase in two-person type designs. With our discussion of ecological validity in mind, it is clear that this trend should continue. However, it may still be beneficial to gain a greater understanding of the potential influence of nuisance variables in social interaction. First, it may be useful to better quantify the degree to which 2D versus 3D stimulus presentation influence different types of social interaction. Additionally, further examination of how gaze differs between realistic and laboratory based social interactions would be of benefit. Some fields have been making use of ecologically valid paradigms for a while (joint action, interpersonal coordination), and others would benefit greatly from mimicking these approaches (e.g., action observation, imitation).

Despite this somewhat idealistic recommendation, it is not always feasible to test two individuals interacting realistically. One-person designs have the benefit of being more easily controlled, and are less open to bias (particularly if the second person is a confederate). Another option is therefore to create stimuli which more accurately reflect social scenarios, but provide a half way point between one- and two-person designs. For example, stimuli could be made to change dynamically, dependent on each participant’s behaviour, rather than simply being a series of static images or video clips. This could also be combined with a move beyond simple key pressing paradigms to more realistic movement or spoken responses. Gaze and the effects of social potential are both closely related to this idea of observation versus interaction. If one-person designs are used, it may be beneficial to more strictly control for gaze and social potential. For example, researchers should make it clear when reporting experiments whether gaze is directly pointed towards objects or persons in every trial, or ensure that there are real life outcomes related to the behaviour of the actor on the screen. Virtual characters provide a strong middle-ground in this instance, though they may suffer from similar limitations as video stimuli, namely a reduction in visual fidelity and some 3D cues (at least outside of a VR setup). However, there is little doubt that the highly controllable nature of virtual characters may provide a solution for the problems of gaze and social potential.

Scanner-based neuroimaging methods such as fMRI or PET can be used to greater effect by using live video feeds to provide more effective interaction between two participants. However, as shown by our research (Reader and Holmes 2015), video latency and temporal variability may be a potential problem in these sorts of designs, especially if differences between 2D and 3D stimuli are a source of nuisance variance. It is imperative that video set-ups are organized in such a way as to bring the interaction as close to the real-life behaviour as possible. New approaches combining virtual characters and neuroimaging may pave the way in these instances. Pfeiffer et al. (2014) provide a strong example of this, particularly considering their control of gaze.

Building on a trend towards two-person neuroscience, so-called hyperscanning methods enable the observation of brain activity in two or more individuals interacting in real-time. Additionally, they provide new insight for a growing interest in measuring brain-to-brain interactions (Hasson et al. 2012). They permit the use of cross-correlations and dyadic data as novel units of analysis, and allow researchers to examine new and interesting questions. In this way, these methods have expanded our knowledge of neural aspects of social interaction (Babiloni and Astolfi 2014; Koike et al. 2015). Whilst hyperscanning in typical scanning environments has been used successfully (e.g., fMRI, Montague et al. 2002), ‘portable’ devices such as electroencephalography (EEG) or functional near-infrared spectroscopy (fNIRS) could provide greater insight into brain activity during ecologically valid experiments, by allowing face-to-face interactions.

In one example, Liu et al. (2015) used fNIRS to examine joint action in a turn-based cooperative and competitive game, allowing the measurement of brain activity in two individuals as they interacted in a highly ecological manner. Also using fNIRS, Jiang et al. (2012) measured the brain activity of two individuals during naturalistic face-to-face communication, back-to-back dialogue or monologue, or face-to-face dialogue. Finally, Delaherche et al. (2014) used EEG in two individuals to derive an automatic measure of imitation during social interaction, showing that EEG is also suitable for these ecological two-person paradigms.

The development of portable hyperscanning methods may lead to a deeper understanding of the way the brain works in realistic social scenarios, and additionally provide new insights into how brain activity in two individuals might interact as a dynamic cause-and-effect process. The integration of a second person could help us to reduce the nuisance variance that may occur as the result of running social interaction studies (typically highly dynamic) in an enclosed, static scanning environment. Of course, this would greatly reduce any potential nuisance effects of visual fidelity, gaze, or reduced social potential. Hari et al. (2015) recently described an excellent framework for the ways in which we might improve our understanding of social interaction using hyperscanning, ultimately suggesting that examining the brain basis for social interaction should step beyond single-participant observation to testing multiple engaged individuals and simultaneous brain recordings. Researchers are beginning to suggest ways in which we can make these dynamic approaches feasible, for example the human dynamic clamp (Dumas et al. 2014).

Finally, potential for more ecologically valid testing in social interaction may also come from outside the research field. One innovative experiment was performed by Ingram et al. (2008) in order to examine the statistical structure of the kinematics of natural hand movements. They provided participants with portable motion-tracking equipment to track the movements of their right hand during day-to-day interactions outside of a laboratory setting. From this data they were able to form a detailed picture of the kinematics and interaction between parts of the hand in real life. Portable methods such as this could be used to provide new insight into naturalistic behaviour outside of the lab. However, considering the complexity of social interactions, serious consideration would be needed in order to properly quantify the variables of interest and the factors affecting them.

Whilst the ways we test social interaction may introduce problems regarding visual fidelity, gaze, and social potential, experimenters are increasingly using new methods that can help to solve these problems. This brief review has hopefully provided insight into the ways in which research in social interaction might be affected by nuisance variance stemming from our choice of methods. Furthermore, we have provided an overview of new ways of approaching experimentation in social interaction, many of which revolve around providing greater interactive potential with a second person. These new approaches may help develop a better understanding of social interaction as it occurs in naturalistic settings.
